# An RNA-seq study in Friedreich ataxia patients identified hsa-miR-148a-3p as a putative prognostic biomarker of the disease

**DOI:** 10.1186/s40246-024-00602-y

**Published:** 2024-05-22

**Authors:** Chiara Vancheri, Andrea Quatrana, Elena Morini, Caterina Mariotti, Alessia Mongelli, Mario Fichera, Alessandra Rufini, Ivano Condò, Roberto Testi, Giuseppe Novelli, Florence Malisan, Francesca Amati

**Affiliations:** 1 Department of Biomedicine and Prevention, Genetics Unit, Tor Vergata University of Rome, Via Montpellier 1, Rome, 00133 Italy; 2 Department of Biomedicine and Prevention, Laboratory of Signal Transduction, Tor Vergata University of Rome, Via Montpellier 1, Rome, 00133 Italy; 3 Unit of Medical Genetics and Neurogenetics, Fondazione IRCCS Istituto Neurologico Carlo Besta, Milan, 20133 Italy; 4 Saint Camillus International University of Health and Medical Sciences, Rome, 00131 Italy; 5 Neuromed Institute, IRCCS, Pozzilli, 86077 Italy; 6 Department of Pharmacology, School of Medicine, University of Nevada, Reno, NV 89557 USA; 7 Present Address: Muscular and Neurodegenerative Diseases Laboratory, Bambino Gesù, Children’s Hospital, IRCCS, Rome, Italy

**Keywords:** microRNA, RNA-seq, Biomarkers, Friedreich ataxia, Frataxin

## Abstract

Friedreich ataxia (FRDA) is a life-threatening hereditary ataxia; its incidence is 1:50,000 individuals in the Caucasian population. A unique therapeutic drug for FRDA, the antioxidant Omaveloxolone, has been recently approved by the US Food and Drug Administration (FDA). FRDA is a multi-systemic neurodegenerative disease; in addition to a progressive neurodegeneration, FRDA is characterized by hypertrophic cardiomyopathy, diabetes mellitus and musculoskeletal deformities. Cardiomyopathy is the predominant cause of premature death. The onset of FRDA typically occurs between the ages of 5 and 15. Given the complexity and heterogeneity of clinical features and the variability of their onset, the identification of biomarkers capable of assessing disease progression and monitoring the efficacy of treatments is essential to facilitate decision making in clinical practice. We conducted an RNA-seq analysis in peripheral blood mononuclear cells from FRDA patients and healthy donors, identifying a signature of small non-coding RNAs (sncRNAs) capable of distinguishing healthy individuals from the majority of FRDA patients. Among the differentially expressed sncRNAs, microRNAs are a class of small non-coding endogenous RNAs that regulate posttranscriptional silencing of target genes. In FRDA plasma samples, hsa-miR-148a-3p resulted significantly upregulated. The analysis of the Receiver Operating Characteristic (ROC) curve, combining the circulating expression levels of hsa-miR-148a-3p and hsa-miR-223-3p (previously identified by our group), revealed an Area Under the Curve (AUC) of 0.86 (95%, Confidence Interval 0.77–0.95; p-value < 0.0001). An *in silico* prediction analysis indicated that the IL6ST gene, an interesting marker of neuroinflammation in FRDA, is a common target gene of both miRNAs. Our findings support the evaluation of combined expression levels of different circulating miRNAs as potent epi-biomarkers in FRDA. Moreover, we found hsa-miR-148a-3p significantly over-expressed in Intermediate and Late-Onset Friedreich Ataxia patients’ group (IOG and LOG, respectively) compared to healthy individuals, indicating it as a putative prognostic biomarker in this pathology.

**Supplementary Information:**

The online version contains supplementary material available at 10.1186/s40246-024-00602-y.

## Introduction

Friedreich Ataxia (FRDA) (OMIM 229300) is a rare and progressive genetic disease that affects the nervous system. In the vast majority of cases, this inherited disease is caused by a homozygous expansion of a trinucleotide GAA repeat sequence within the first intron of the *FXN* gene, which encodes a protein called frataxin. This mutation severely affects gene transcription, causing a deficiency of the frataxin protein [1]. Frataxin plays a crucial role in regulating iron metabolism within cells and in energy production. Its deficiency results in a toxic buildup of iron in nerve cells, particularly the dorsal root ganglia and cerebellum, leading to progressive damage to the central and peripheral nervous systems [2, 3]. Frataxin levels are crucial not only for cell survival, but also for stress-management responses [4–6].

FRDA affects 1:50,000 individuals, making it the most prevalent hereditary ataxia in the Caucasian population [7]. The primary manifestation of FRDA is neurological, with symptoms that include progressive loss of coordination and balance, muscle weakness, and sensory impairment. Individuals with this condition often experience difficulty walking due to gait abnormalities, impaired speech, and coordination problems that worsen over time [8]. Although the rate of progression can vary between individuals, most people with FRDA eventually require mobility aids, such as wheelchairs. In addition to neurological symptoms, FRDA can also affect various other organs and systems in the body. Cardiac abnormalities, such as hypertrophic cardiomyopathy, are one of the major complications associated with this condition and are the predominant cause of premature death [9]. Some individuals may also develop diabetes mellitus due to degeneration of pancreatic beta cells that produce insulin [10].

The onset of FRDA typically occurs in childhood or adolescence, usually between the ages of 5 and 15, although it can also manifest in adulthood [11]. Patients who develop clinical signs after the ages of 25 and 40 are defined as Late-Onset (LOFA) and Very Late-Onset (VLOFA) patients respectively. Since the disease tends to progress gradually over the years, symptoms worsen with time, a milder phenotype, slower progression of the disease and more variable signs and symptoms characterize LOFA and VLOFA patients [12, 13]. Usually, age at onset and disease severity correlate with the length of the GAA expansion, with longer expansions associated with earlier manifestation of symptoms and a more rapid progression.

Currently, limited therapeutic options are available to patients. The FDA has approved the Nrf2 activator Omaveloxolone in 2023, as the first treatment for FRDA [14, 15]. In addition, several different therapeutic approaches have been explored to manage symptoms and potentially slow the progression of the disease [16–18]. Although these therapeutic opportunities show promise, the path to final approval can be difficult [19, 20]. Numerous clinical studies are currently underway, but there are challenges to consider when developing a trial for a rare disease such as Friedreich ataxia. Difficulties include limited patient population, disease heterogeneity, and limited natural history data, which make it difficult to establish critical endpoints and identify reliable outcome measures [21].

In recent years, epigenetic mechanisms have emerged as important players in the development of complex pathological phenotypes [22, 23]. Among the best-studied epigenetic mechanisms, microRNAs (miRNAs) are a group of small non-coding RNA molecules ($\sim$22 nucleotides in length) that regulate gene expression at post-transcriptional level by binding specific mRNA targets. Many studies have demonstrated that miRNAs are critically involved in important biological processes in healthy and diseased conditions, including cancer, Alzheimer’s disease, cardiovascular diseases, viral infections and diabetes [24–29]. Dysregulated expression of specific miRNAs can contribute to disease progression by affecting critical signaling pathways and gene networks. In the context of neurodegenerative diseases, miRNAs have often been found to be dysregulated in affected brain regions in Alzheimer disease, Parkinson disease [30, 31] and amyotrophic lateral sclerosis patients [30]. These dysregulated miRNAs can influence the expression of genes involved in neuronal survival, oxidative stress, inflammation, protein aggregation, and other pathological processes associated with neurodegenerative conditions [31].

Currently, the possible impact of FRDA-related epigenetic alterations, including miRNA-based regulatory mechanisms, is being extensively investigated. A study described the presence of a single nucleotide polymorphism (SNP) that creates a binding site for hsa-mir-124-3p in the 3’UTR of the FXN gene, further reducing frataxin expression in patients [32]. Hsa-miR-886-3p was found to be elevated in FRDA patients and to negatively regulate frataxin transcription [33]. A SNP that alters the miR-155 binding site in the angiotensin II type 1 receptor gene (AGTR1) and that potentially contributes to cardiac manifestations of the disease, has been identified in FRDA patients [34].

In addition, the presence of miRNAs in circulating blood has raised the possibility that they could be putative genomic biomarkers for various diseases. The potential role of circulating miRNA as biomarkers in monitoring disease progression and therapeutic intervention outcome in FRDA is also being explored. Previous studies show that FRDA patients have an altered miRNA expression pattern [35–37]. In this context, hsa-miR-323a-3p was found to be significantly elevated in plasma samples from FRDA patients with cardiac complications and was therefore proposed as a biomarker for the diagnosis of cardiomyopathy in FRDA [38]. Our group identified hsa-miR-223-3p, upregulated in plasma from FRDA patients, as another potential biomarker of cardiac disease in FRDA [35]. In fact, we found a significant positive correlation between hsa-miR-223-3p expression level and cardiac parameters (IVS, interventricular septal wall thickness, and LPW, left ventricular posterior wall thickness) in typical FRDA patients (onset < 25 years) [35]. These data suggested that an increase in circulating hsa-miR-223-3p expression might be associated with a more severe cardiac FRDA phenotype and propose that hsa-miR-223-3p could be studied as a biomarker of cardiac disease progression in FRDA [35].

Identifying a miRNA profile that occurs during the natural history of FRDA disease could be of relevance as miRNA levels change with disease progression and/or pharmacological interventions. Moreover, the identification of new miRNAs could contribute to the design of novel therapeutic strategies and improve clinical decisions.

The aim of this study was to find a miRNA signature associated with the progression of the disease. Thus, we first conducted an RNA sequencing study by NGS (Next Generation Sequencing) in peripheral blood mononuclear cells (PBMCs) of a selected group of FRDA patients (*n* = 12; grouped based on the onset of clinical signs and severity) and a group of healthy donors of the same age and sex (CTRL; *n* = 4). Our RNA-seq study evaluated the expression level of circulating small non-coding RNAs (sncRNA), i.e. not only miRNAs, but also snoRNAs, snRNAs and piRNAs. Second, we analysed differentially expressed (DE) sncRNAs identified by RNA-seq in all enrolled samples (*n* = 61).

Hsa-miR-148a-3p resulted significantly upregulated in plasma samples from FRDA patients (*p* < 0.05); of interest, its expression level was increased in intermediate and late onset patients (*p* < 0.05). An *in silico* prediction analysis of both hsa-miR-223-3p and hsa-miR-148a-3p, indicated the IL6ST (Interleukin 6 Cytokine Family Signal Transducer) gene, a previously identified marker of neuroinflammation in FRDA [39], as a common target gene.

## Results

### Study population

For this expression study, we enrolled 61 subjects, 23 healthy controls and 38 FRDA patients. The clinical data of the 61 subjects studied are summarized and reported in our previous studies [35, 40].

### RNA-sequencing

We aimed to identify new circulating miRNAs in the progression of FRDA disease. Consequently, we first performed a miRNA-seq analysis on PBMCs isolated from 12 out of 38 FRDA patients and 4 out of 23 age-matched healthy controls (Table 1). The 12 FRDA patients were chosen among those with an early onset of neurological symptoms (< 14 years) and a worse course of the disease (named early-onset group, EOG, *n* = 4); those with an intermediate onset of neurological symptoms (14–25 years) and a slight disease course (intermediate-onset group, IOG, *n* = 4) and those with a late onset of neurological symptoms (> 25 years) and a slow disease course (late-onset group, LOG, *n* = 4) (Table 1).

**Table 1:** Principal data of the 12 FRDA patients (PAT) and 4 healthy subjects (CTRL) analyzed by RNA-seq

	FRDA Patients	Age at onset	Gender	Age at blood collection
EOG	PAT 1	10	M	33
	PAT 2	14	M	26
	PAT 3	12	M	38
	PAT 4	7	F	37
IOG	PAT 5	15	F	28
	PAT 6	20	M	33
	PAT 7	20	F	46
	PAT 8	16	M	28
LOG	PAT 9	24	F	49
	PAT 10	35	F	52
	PAT 11	40	F	53
	PAT 12	31	F	43
CTRL	CTRL1	-	F	47
	CTRL2	-	M	28
	CTRL3	-	M	29
	CTRL4	-	F	39

The mean age of CTRL (*n* = 4) was 35 ± 9 years while the mean age of all FRDA patients (*n* = 12) was 38 ± 10 years. The RNA-seq was performed in collaboration with Biodiversa S.r.l. (http://www.biodiversa.it). Based on the average expression of the small non coding RNAs (sncRNAs) across all samples, the top 5% expressed sncRNAs were identified. The list of sncRNAs, their expression across the samples as well as the average, the rank and percentage of top expressed genes are reported in the Supplementary Table 1 (Table S1).

**Fig. 1:**
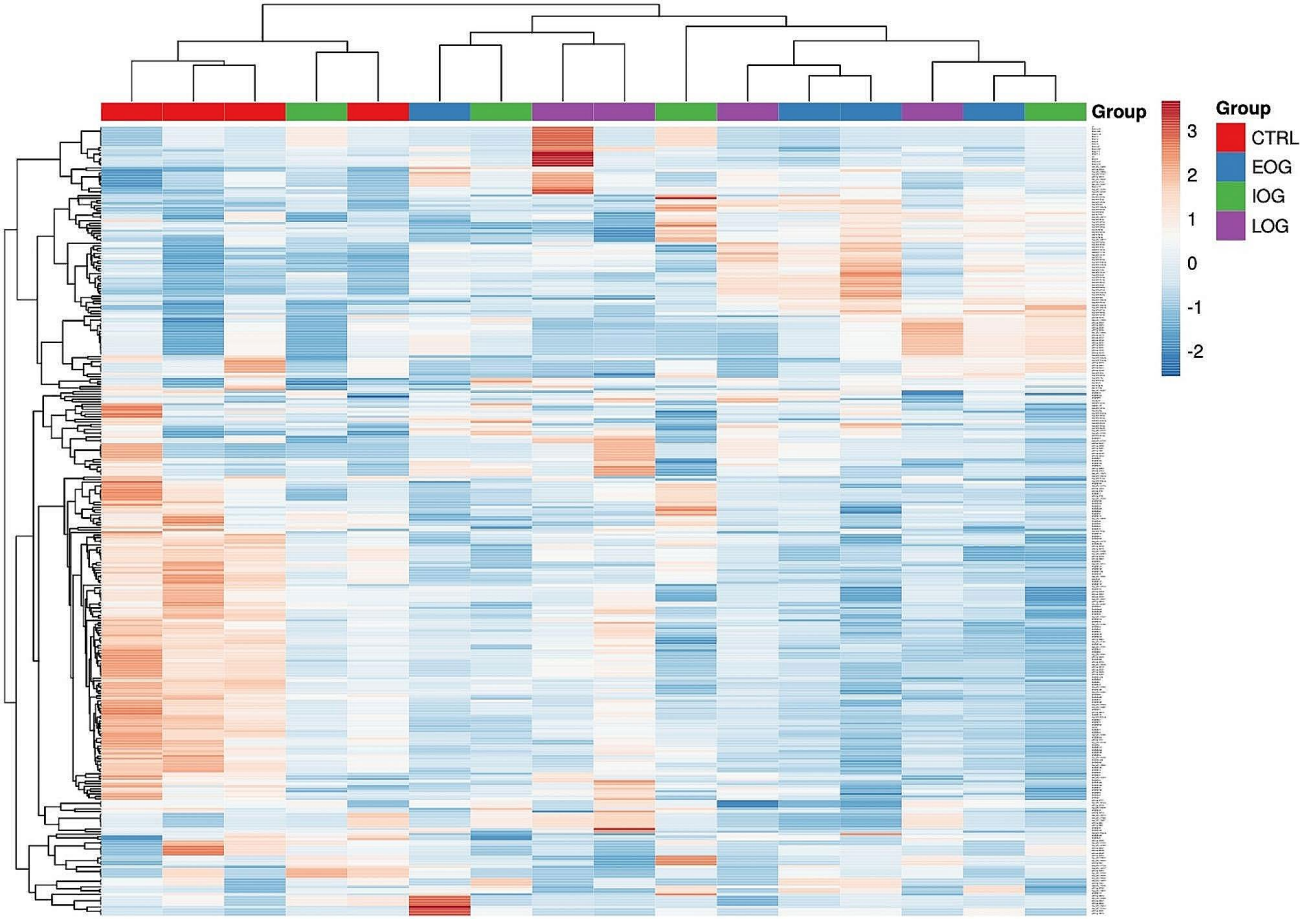
Heatmap illustrating the expression profiles (scaled TMM) of the top 5% expressed sncRNAs detected in the 16 samples sequenced. The bar on the left visualizes the expression level of the top 5% expressed sncRNAs; in orange-red the upregulated sncRNAs, in blue the downregulated ones. Each group is labeled with a distinct color (i.e. CTRL group = red)

A hierarchical clustering algorithm (HCA) consistently detected a significant differential expression of several sncRNAs (*p* < 0.05) among the analyzed patients. A sncRNAs signature common to CTRL group is evident and able to distinguish this group from the majority of FRDA patients (Fig. 1).

**Fig. 2:**
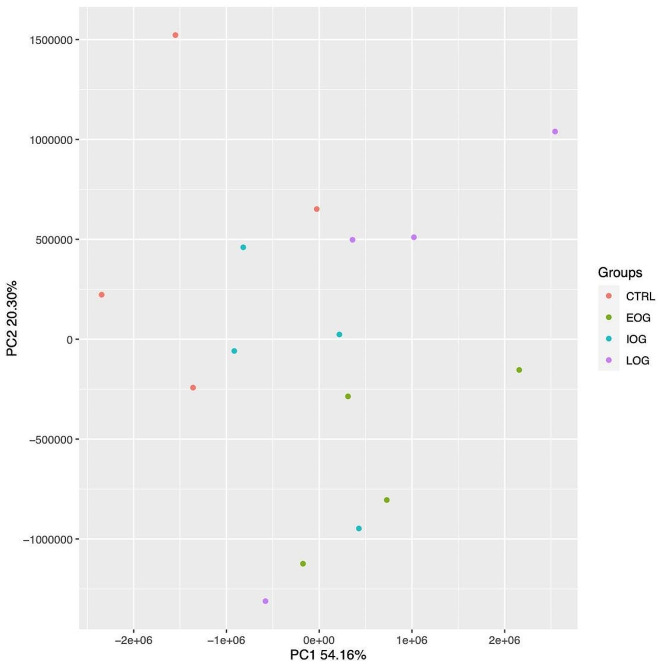
Principal Component Analysis (PCA) conducted on the normalized RNA expression values of all samples. X- and Y-axes show the PC1 and PC2, respectively, with the amount of variance explained by each component that is reported. Each point in the plot represents a sample, dots of the same colors are replicates of the same group (i.e.green dots = EOG patients’ group)

A Principal Component Analysis (PCA) was performed to evaluate the clustering of our samples (Fig. 2).

Even if the replicates of each group showed variability, we performed a standard analysis using all samples. All FRDA patients’ groups were pairwise compared with CTRL group; in addition, we compared each patients group, i.e. EOG vs IOG, EOG vs LOG, IOG vs LOG.

The EOG vs CTRL comparison produced 228 differentially expressed sncRNAs (DE-sncRNAs), which are 151 miRNAs, 33 snoRNAs, 16 snRNA and 28 piRNAs (Table S2). Supplementary Figure S1 shows a heat map with the profile of these differentially expressed sncRNAs.

The IOG vs CTRL comparison evidenced 69 differentially expressed sncRNAs which are 51 miRNAs, 3 snoRNAs, 7 snRNAs and 8 piRNAs (Table S3). The heatmap with the profile of these DE-sncRNAs is depicted in Supplementary Fig. 2 (Figure S2).

The LOG vs CTRL comparison revealed 76 differentially expressed sncRNAs, specifically 59 miRNAs, 6 snoRNAs, 2 snRNAs and 9 piRNAs (Table S4). Supplementary Figure S3 indicates a heatmap with the profile of these DE-sncRNAs (Figure S3).

The comparisons among the different FRDA patients’ group resulted in 8 DE-sncRNAs in EOG vs IOG and 3 DE-sncRNAs in EOG vs LOG (Table S5). However, all these DE-sncRNAs were piRNAs; none differentially expressed miRNA was identified among the different FRDA patients’ group. Furthermore, the IOG vs LOG comparison did not produce statistically significant results.

To identify miRNAs potentially involved in Friedreich ataxia pathogenesis, we focused our attention on DE-miRNAs in comparisons between FRDA patients and CTRL. We selected four DE-miRNAs: hsa-miR-143-3p and hsa-miR-199b-5p that are upregulated in all FRDA patients’ groups compared to healthy subjects (CTRL); hsa-miR-93-5p that is upregulated in the comparisons EOG vs CTRL and LOG vs CTRL and hsa-miR-148a-3p which is the only miRNAs over-expressed in the comparison EOG vs CTRL and IOG vs CTRL (Table 2).

**Table 2:** List of the most significant DE-miRNAs in the comparison of FRDA patients vs CTRL

	MiRBase ID	logFC	logCPM	LR	PValue	FDR
EOG	hsa-miR-143-3p	4.24	9.08	79.32	5.28E-19	1.22E-15
	*hsa-miR-223-3p*	*4.22*	*12.04*	*79.13*	*5.82E-19*	*1.22E-15*
	hsa-miR-199b-5p	3.24	7.58	56.51	5.58E-14	2.93E-11
	hsa-miR-93-5p	2.00	9.20	28.35	1.01E-07	2.12E-05
	hsa-miR-148a-3p	1.63	11.67	16.26	5.51E-05	0.0047
	hsa-miR-16-5p	1.57	8.97	18.72	1.51E-05	0.0017
	hsa-miR-342-5p	-1.50	4.45	15.85	6.84E-05	0.005
IOG	hsa-miR-143-3p	3.53	9.08	58.67	1.87E-14	1.18E-10
	*hsa-miR-223-3p*	*3.01*	*12.04*	*45.00*	*1.97E-11*	*2.48E-08*
	hsa-miR-199b-5p	2.24	7.58	29.46	5.69E-08	2.99E-05
	hsa-miR-148a-3p	1.60	11.67	15.65	7.61E-05	0.01
LOG	*hsa-miR-223-3p*	*3.45*	*12.04*	*56.80*	*4.83E-14*	*3.04E-10*
	hsa-miR-143-3p	3.34	9.08	53.49	2.60E-13	8.20E-10
	hsa-miR-199b-5p	2.59	7.58	38.15	6.53E-10	4.11E-07
	hsa-miR-93-5p	1.29	9.20	12.25	0.00046	0.042
	hsa-miR-16-5p	1.24	8.97	11.83	0.00058	0.048
	hsa-miR-342-5p	-1.42	4.45	14.29	0.00016	0.018

Interestingly, among the DE-miRNAs common to all FRDA patients’ groups we confirmed the upregulation of hsa-miR-223-3p (in italics in Table 2) that we have previously identified as overexpressed in plasma from FRDA patients and significantly correlated with cardiac parameters (IVS and LPW) in FRDA patients [35].

### Hsa-miR-148-3p is upregulated in FRDA plasma

Since circulating plasma miRNAs can be easily accessible through non-invasive procedures, they represent invaluable biomarkers for disease diagnosis, prognosis, and therapy monitoring. We evaluated the four DE-miRNAs identified from RNA-seq (hsa-miR-143-3p, hsa-miR-199b-5p, hsa-miR-93-5p and hsa-miR-148a-3p) on RNA extracted from plasma samples of all case study (38 FRDA patients and 23 CTRL subjects).

**Fig. 3:**
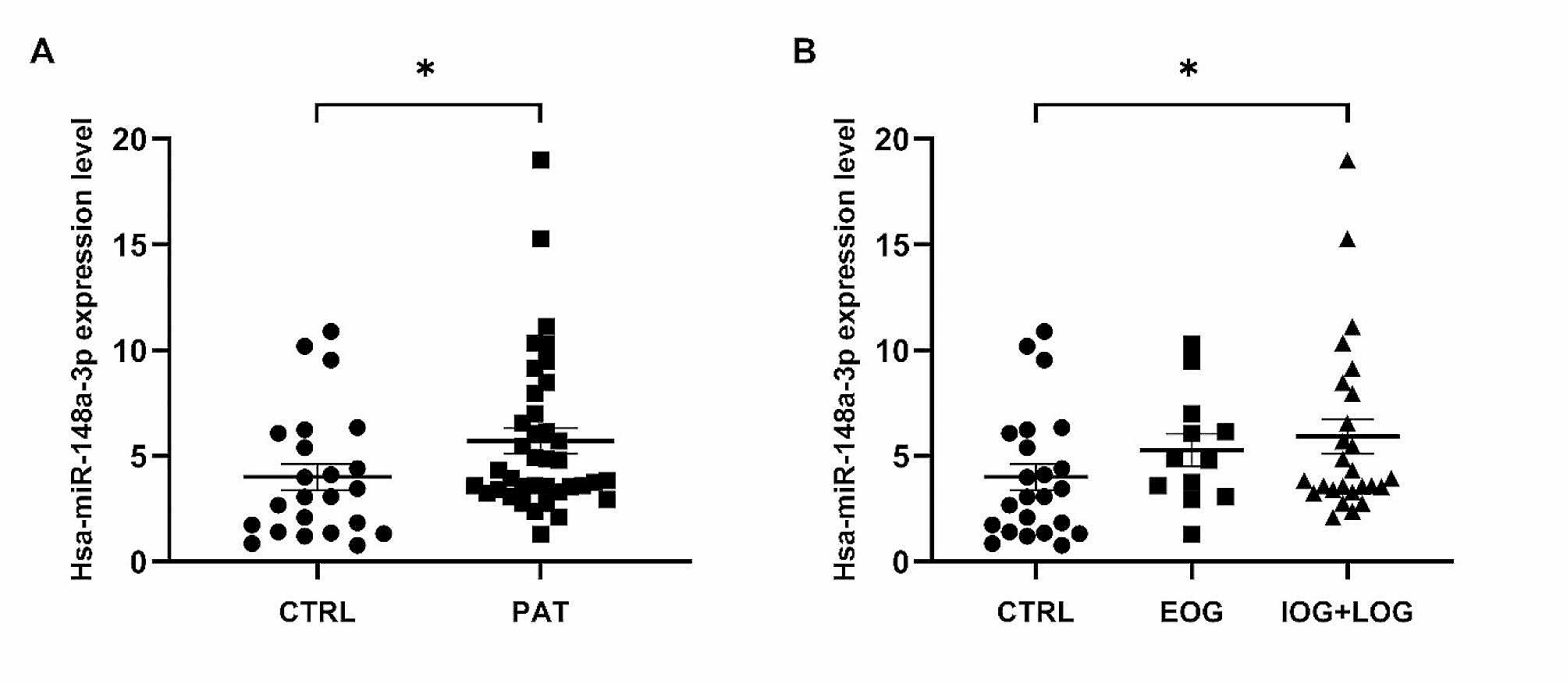
Hsa-miR-148a-3p expression level in plasma of all cases studied. (**A**) Comparison of control subjects (CTRL) and all FRDA patients (PAT). Mann-Whitney test. *p < 0.05. (**B**) Hsa-miR-148a-3p expression level in plasma of intermediate and late onset group (IOG + LOG) patients vs control subjects (CTRL). Mann-Whitney test. *p < 0.05

Among the miRNAs analyzed, hsa-miR-148a-3p resulted the only significantly dysregulated miRNA in plasma samples of all FRDA patients (*p* < 0.05; Fig. 3A). Interestingly, we observed a significant increase in IOG + LOG patients’ group when this group was compared to the control group (CTRL) using the non-parametric test Mann-Whitney, which compares only two different experimental groups (*p* < 0.05) (Fig. 3B).

### ROC curve and correlation analysis of hsa-miR-148a-3p

**Fig. 4:**
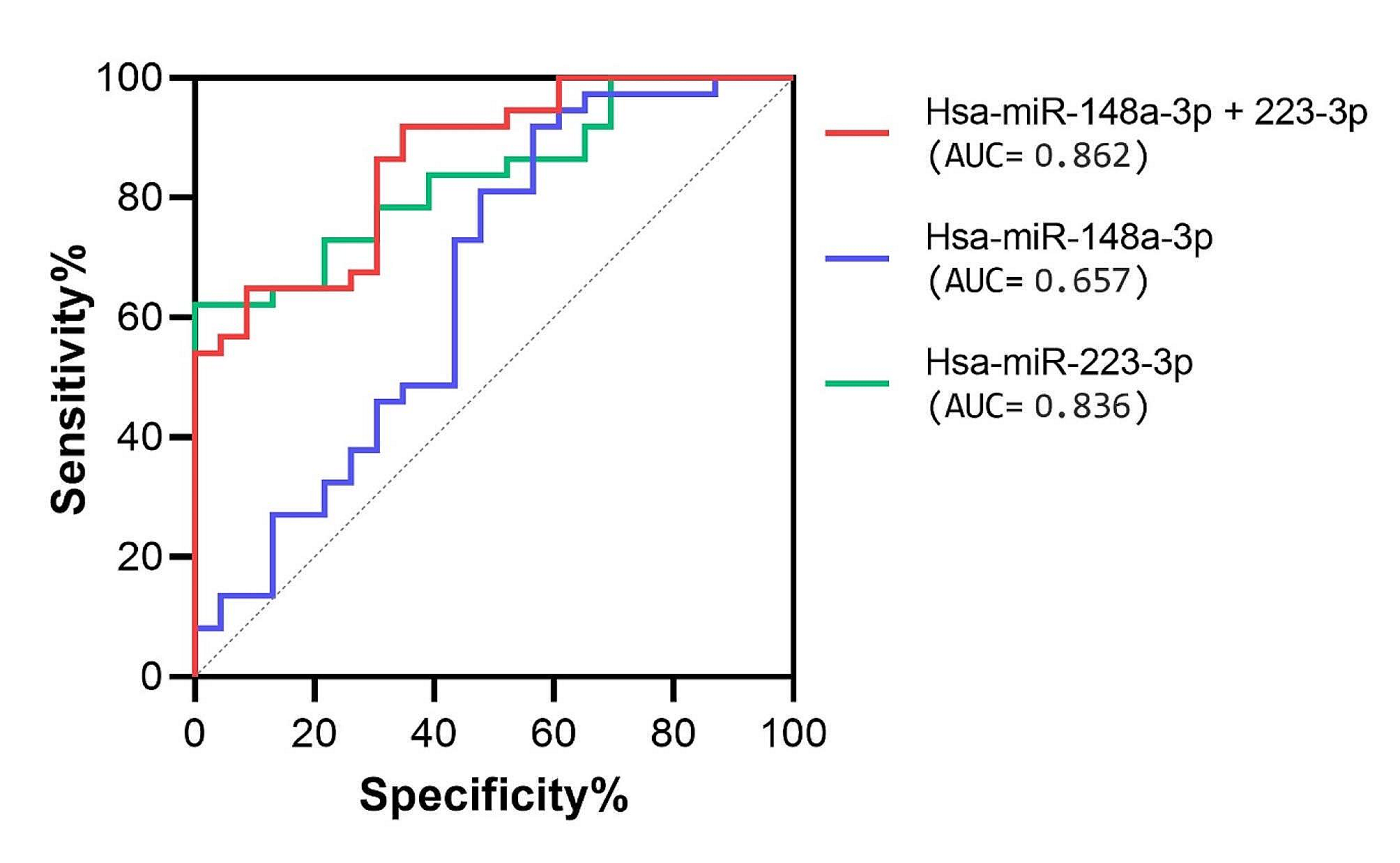
ROC curve analysis of hsa-miR-148a-3p, hsa-miR223-3p and the combination of the two for the discrimination of FRDA patients

The ROC curve analysis is a statistical method used also to assess the diagnostic ability of a test or marker to discriminate between subjects who present a given disease and those who do not. We performed a ROC curve analysis to investigate the potential capacity of miR-148a-3p to distinguish FRDA patients from control subjects. A ROC curve of hsa-miR-148a-3p, obtained when plotting control subjects against FRDA patients, indicates a moderate power for discrimination of FRDA patients from CTRL subjects (AUC = 0.66, 95% confidence interval 0.51–0.81, *p* < 0.05) (Fig. 4).

No correlation was found among the expression level of hsa-miR-148a-3p and the available clinical data of FRDA patients. Moreover, no correlation was found between hsa-miR-148a-3p levels and the age of FRDA patients at sample collection. In addition, we analyzed by Pearson correlation test, the relationship between the expression profile of hsa-miR-148a-3p and the expression levels of the other circulating DE-miRNAs but no significant correlation was found (data not shown).

### Combined ROC curve analysis of miR-223-3p and miR-148a-3p

We tested a predictive model combining the circulating expression levels of hsa-miR-148a-3p and hsa-miR-223-3p, previously identified as over-expressed in the same FRDA group [35, Fig. 4], to evaluate the capacity of these two upregulated miRNAs to discriminate FRDA patients from healthy subjects. Analysis of the combined ROC curve revealed that the area under the ROC curve (AUC) was 0.86 with a 95% confidence interval of 0.77 to 0.95 (p-value < 0.0001) (Fig. 4).

### In silico prediction analysis on putative target genes common to hsa-miR-223-3p and hsa-miR-148a-3p

Based on the high combined diagnostic value of hsa-miR-223-3p and hsa-miR-148a-3p expression levels, we performed an *in silico* analysis to search for common target genes, i.e. putative genes regulated by both these miRNAs. We used the prediction software DIANA-mirPath [41]. DIANA-mirPath is a miRNA pathway analysis web-server, which can utilize predicted miRNA targets (in CDS or 3’-UTR regions) provided by the DIANA-microT-CDS algorithm or even experimentally validated miRNA interactions derived from DIANA-TarBase.

Interestingly, IL6ST (Interleukin 6 Cytokine Family Signal Transducer) gene that encodes a signal transducer shared by many cytokines, including interleukin 6 (IL-6), ciliary neurotrophic factor (CNTF), leukemia inhibitory factor (LIF), and oncostatin M (OSM), resulted the unique putative target gene of both hsa-miR-223-3p and hsa-miR-148a-3p.

## Discussion

Heterogeneity in FRDA clinical presentation, disease severity, tissue involvement, and type of FXN mutation render mandatory to select the best and most robust outcome biomarker to track disease progression.

In this study, we performed an RNA-seq analysis by NGS to analyze the expression level of circulating small non-coding RNAs (sncRNAs) in PBMCs from FRDA patients. A signature of sncRNA able to distinguish control (CTRL) group from the majority of FRDA patients was detected with a hierarchical clustering algorithm (HCA, Fig. 1). Interestingly, a large number of differentially expressed sncRNAs (DE-sncRNAs) and of differentially expressed miRNAs (DE-miRNAs) were detected in the EOG patients vs CTRL group comparison, indicating a more pronounced perturbation of sncRNAs profile in the more severe form of the disease.

Our study identified a significant upregulation of hsa-miR-148a-3p in FRDA plasma samples (Fig. 3A). Noteworthy, hsa-miR-148a-3p increase was also significant when the intermediate and late onset FRDA patient’s groups (IOG + LOG) were compared with the control group (Fig. 3B), suggesting a possible involvement of this miRNA in milder forms of the disease. In addition, analysis of ROC curves combining the upregulated expression values of hsa-miR-148a-3p and hsa-miR-223-3p, previously identified by our group [35] and confirmed upregulated in this study, revealed an AUC of 0.86 with 95% confidence interval of 0.77 to 0.95 (p-value < 0.0001) (Fig. 4). This AUC value indicates that the combined evaluation of hsa-miR-148a-3p and hsa-miR-223-3p has a strong diagnostic accuracy and, therefore, potential clinical utility [42]. The combination of two or more putative biomarkers could help explain the complex pathogenic mechanisms of FRDA rather than a single biomarker that is unlikely to dictate the complicated evolutionary process at the systemic level.

Our result is in agreement with a previous report describing hsa-miR-148a-3p upregulation in FRDA patients fibroblast cells [43]. Moreover, hsa-miR-148a-3p differential expression is linked to several diseases, such as tumors, cardiovascular diseases (CVD), neurological disorders. In several cancers, hsa-miR-148a-3p has been reported as a tumor inhibitor [44–47]. In CVD, hsa-miR-148a-3p regulates lipid metabolism, is upregulated in atherosclerosis patients, promotes the proliferation and migration of endothelial cells and might play a protective role in atrial fibrillation (AF) [48–50]. Noteworthy, cardiac expression of miR-148a is altered in different subtypes of human and mouse forms of heart failure; high miR-148a expression is observed in human and mouse forms of concentric hypertrophy and decreased miR-148a expression is detected in forms of dilated cardiomyopathy [51]. Hsa-miR-148a-3p is involved in neurological disorders such as Alzheimer’s Disease (AD) and Parkinson’s disease. In AD, its upregulation reduces tau hyperphosphorylation and protects neuronal cells against Aβ-induced injury by targeting p35 and PTEN in brain [52]. In Parkinson’s disease miR-148a-3p is regulated by lithium that exhibits significant neuroprotective effects [53]. Interestingly, the cellular localization of miR-148a-3p in vivo and in vitro was found in neurons and astrocytes and its downregulation inhibited apoptosis of hippocampal neurons after kainic acid (KA) induced through the PI3K/Akt signaling pathway [54].

A combination of our results and previous literature suggests that hsa-miR148a-3p may play a functional role in the FRDA clinical presentation and disease progression. This, of course, requires further experimental confirmation in a larger number of FRDA patients and in FRDA cells and animal models.

Among the DE-miRNAs identified in this RNA-seq study, we observed a trend for the increase of hsa-miR-199b-5p in FRDA patient group, although not statistically significant (Figure S4A). Interestingly, hsa-miR-199b-5p upregulation was significant in the Late-Onset FRDA patients’ group (LOG) compared to control group using the Mann-Whitney test (*p* < 0.01; Figure S4B). Abnormal expression of miR-199 may be involved in the pathophysiology of human epilepsy [55] and a role of hsa-miR-199b-5p in heart diseases has been suggested. The miR-199 family has been shown to participate in pathological cardiac hypertrophy [56, 58]. A cardiomyocyte-specific miR-199-sponge transgenic mouse model developed physiological cardiac hypertrophy that uncovers a surprising role for endogenous miR-199 in maintaining cardiac homeostasis [56–58].

Our *in silico* prediction analysis of common target genes of both hsa-miR-148a-3p and hsa-miR-223-3p, i.e., significant upregulated miRNAs in FRDA, indicated that the IL6ST gene is a strong candidate. IL6ST encodes the glycoprotein gp130 that acts as the interleukin-6 (IL-6) signal transducing receptor subunit for the entire IL-6 family of cytokines [59]. Cytokine receptor gp130, is a key component of the interleukin 6 receptor (IL-6R)/IL6ST complex in the microglial membrane. Signaling of gp130 has essential homeostatic and protective roles, for example in inflammation, metabolism or neural development [60–63]. Gp130 homodimerizes only if activated by IL-6/IL-6R complexes and initiates downstream Jak/Stat signaling, which is essential for innate immunity as well as neuronal functions [62]. Interestingly, frataxin-deficient lymphoblasts from Friedreich’s patients overexpress IL-6 and other inflammatory transcripts, including TNF, and these inflammatory changes were rescued by frataxin transfection [64]. Moreover, increased plasma levels of IL-6 were found in FRDA patients showing neuroinflammation in the cerebellum and brainstem [39]. A dysregulated expression of IL6ST, mediated by hsa-miR-148a-3p and hsa-miR-223-3p, could affect the downstream molecular signaling pathway associated with IL6ST, that is, the effects of IL-6.

Our results might be further confirmed in other FRDA patient’s groups to definitely determine hsa-miR-148a-3p and hsa-miR-223-3p as biomarkers of disease in Friedreich ataxia and, in the future, putative therapeutic targets.

## Conclusions

Our findings support the evaluation of combined expression levels of different circulating miRNAs as potent epi-biomarkers in FRDA. Furthermore, we identified hsa-miR-148a-3p as a miRNA particularly expressed in intermediate and late-onset patients, indicating it as a potential prognostic biomarker in this pathology. Finally, we *in silico* identified a putative target gene, IL6ST, of both miRNAs upregulated in FRDA patients, thus suggesting a mechanistic role of these miRNAs in the inflammation mechanism underlying FRDA pathogenesis.

## Materials and methods

### Patient’s recruitment and samples collection

We studied 61 individuals, including 38 FRDA patients and 23 control subjects (CTRL group). The mean age of FRDA patients’ group was 42 years ± 13, while in healthy controls the mean age was 41 years ± 13. A more detailed clinical description of these patients is available in Tiano et al. [40] and in Quatrana et al. [35]. Briefly, the FRDA group was constituted by patients with typical age of onset of neurological symptoms before 25 years, and patients with late-onset disease (LOFA, age at onset ≥ 25 years). FRDA patients with typical onset had the shorter allele > 750 GAA, and LOFA patients had the shorter allele with 150–350 GAA. For the RNA-sequencing (RNA-seq) study, we decided to further divide our FRDA patients into 3 groups: (1) those with an early onset of neurological symptoms (< 14 years) and a worse course of the disease (named early-onset group, EOG); (2) those with an intermediate onset of neurological symptoms (14–25 years) and a slight disease course (intermediate-onset group, IOG) and (3) those with a late onset of neurological symptoms (> 25 years) and a slow disease course (late-onset group, LOG).

The FRDA patients we enrolled came from three different clinical centers: Policlinico Tor Vergata-Fondazione PTV (Rome). La Sapienza University of Rome and Istituto Neurologico Carlo Besta (Milan).

This project was approved by the Ethical Committee of Policlinico Tor Vergata-Fondazione PTV (n. 47/16 and n.56). All the enrolled patients gave informed consent prior to the inclusion in the study. All the principles outlined in the Helsinki Declaration of 1975, as revised in 2013, have been followed in all the assays involving human subjects during the current study.

### PBMCs isolation, total RNA extraction and reverse transcription

RNA-seq study was performed on RNA obtained from PBMCs. PBMCs from a peripheral blood sample (9 mL) of each FRDA patient and CTRL subject were isolated using density-gradient medium. About 4 mL of fresh blood was gently layered on equal volume of Ficoll-Paque Plus® (GE Healthcare, Little Chalfont, UK) and then centrifuged at 400xg for 40 min at RT without brake. The mononuclear cell layer was transferred in a new tube and washed two times with Phosphate Buffered Saline (PBS). The cell pellet was resuspended in 1 mL of Trizol® (Ambion, Waltham, MA, USA) and stored at -80 °C. Total RNA, including miRNAs fraction, was extracted from these cell pellets according to manufacturer’s instructions (Trizol®, Ambion, Waltham, MA, USA). RNA concentration was evaluated by using a NanoDrop ND-1000 Spectrophotometer (Euro-Clone), whereas RNA quality was checked on agarose gel 1%.

Quantitative real time PCR (qRT-PCR) was conducted on miRNA fraction. To isolate miRNAs fraction, 50ng of total RNA from PBMCs was reverse transcribed into cDNA using the miRCURY LNA RT Kit (QIAGEN) following the manufacturer’s instructions.

### Plasma isolation. miRNAs extraction and reverse transcription

Plasma samples have been isolated by whole blood using Ficoll-Plaque Plus® (GE Healthcare, Little Chalfont, UK) according to manufacturer’s instructions. Briefly, we added 3 mL of Ficoll-Paque Plus to a centrifuge tube and then carefully layered 4 mL of diluted blood sample (4 mL). After a centrifugation step at 400×g for 40 min at RT, we drew off the upper layer of plasma using a clean Pasteur pipette. Subsequently, plasma samples were centrifuged for 10 min at 16,000×g in order to remove additional nucleic acids attached to cell debris and then stored at − 80° C.

Total RNA, including microRNAs, was extracted from 200µL of plasma using miRNeasy Serum/Plasma Kit (QIAGEN) according to the manufacturer’s instructions. Sixteen µL of total RNA has been reverse transcribed into cDNA using the miRCURY LNA RT Kit (QIAGEN) following the manufacturer’s instruction.

### MiRNA sequencing and data processing

The RNA-seq study has been performed in collaboration with Biodiversa s.r.l. (http://www.biodiversa.it). One µg of fresh RNA extracted from PBMCs of four subjects from each group (EOG, IOG, LOG and CTRL), matched for age and sex (Table 1), have been sequenced to identify different expression level of sncRNAs and in particular a FRDA miRNA signature.

The quality of the reads was assessed with the software FASTQC, and then a trimming step was performed to remove adapters and low quality bases from the reads. The following parameters were used: the minimum length was set to 15 bp and the quality score to 25. The software BBDuk was used for this scope. On average 95% of the reads passed the quality control. The high quality reads from human samples were aligned against the Homo sapiens genome (GRCh38 from Ensembl. https://www.ensembl.org/index.html) with STAR aligner (version 2.7.1a). On average 99% of the reads mapped on the genome and 42% mapped uniquely. The identification and expression quantification of small non-coding RNAs (sncRNA) such as miRNAs, snoRNAs, snRNAs and piRNAs, were performed with the COMPSRA pipeline. Based on the average expression of the genes across all the samples, the top 5% expressed small non-coding RNAs were identified. The statistical analysis was performed with edgeR applying the TMM normalization. Only sncRNAs showing an FDR (false discovery rate) < = 0.05 were considered statistically significant. RNA-Seq data were deposited to Gene Expression Omnibus (GEO) under accession number GSE243874 (https://www.ncbi.nlm.nih.gov/geo/query/acc.cgi?acc=GSE243874).

### MiRNAs expression analysis by qRT-PCR

Among the DE-miRNAs, we analyzed only those having the following criteria: (1) a higher expression level across all the samples (i.e. these miRNAs must be present in the top 5% expressed sncRNA list, Table S1); (2) a common behavior among at least two FRDA group; (3) a FDR (false discovery rate) value < 0.05. MiRNAs expression analysis on plasma samples was carried out using the ABI7500 Fast Real-time PCR System (Life Technologies) in triplicate from at least two independent experiments (*n* = 6) using miRCURY LNA Sybr Green PCR Kit (QIAGEN) and a specific primer assay for hsa-miR-199b-5p (hsa-miR-199b-5p GeneGlobe ID - YP00204152), hsa-miR-143-3p (hsa-miR-143-3p GeneGlobe ID - YP00205992), hsa-miR-148a-3p (hsa-miR-148a-3p GeneGlobe ID - YP00205867) and hsa-miR-93-5p (hsa-miR-93-5p GeneGlobe ID - YP00204715).

Hsa-miR-320c (hsa-miR-320c GeneGlobe ID-YP00205706 QIAGEN) and hsa-miR-598-3p (hsa-miR-598-3p GeneGlobe ID-YP00204320 QIAGEN) were used as endogenous controls. Indeed, hsa-miR-320c and hsa-miR-598-3p were commonly expressed in PBMCs of all analyzed groups and showed an FDR of 1 (therefore not statistically significant) and a stable expression among samples. Moreover, we verified that in the literature these two miRNAs were not involved in ataxias (not only in the Friedreich ataxia) and in cardiovascular diseases. Data analysis was performed using the comparative Ct method quantification (2^−∆Ct^ method) [35].

### In silico prediction analysis of target genes

We performed an *in silico* analysis using the DIANA-mirPath database (https://diana-lab.e-ce.uth.gr/app/miRPathv4) to identify common target genes, i.e. putative genes regulated both by hsa-miR-148a-3p and by hsa-miR-223-3p. DIANA-miRPath is a web server for miRNA pathway analysis, providing accurate statistics while able to accommodate advanced pipelines. MiRPath can utilize predicted miRNA targets (in CDS or 3’-UTR regions) provided by the DIANA-microT-CDS algorithm or even experimentally validated miRNA interactions derived from DIANA-TarBase v6.0. These interactions (predicted and/or validated) can be subsequently combined with sophisticated merging and meta-analysis algorithms.

### Statistical analysis

All the statistical analysis in the sncRNA sequencing study were performed with the package edgeR. Experimental data were statistically analyzed using SPSS 26 (SPSS Inc., Chicago, IL, USA) and GraphPad Prism 9 (GraphPad Software Inc., La Jolla, CA, USA).

The distribution of expression data was analyzed by Kolmogorov–Smirnov test. Mann–Whitney test and Kruskal–Wallis test were used for data analysis when appropriate. For both parametric and non-parametric distribution, expression data are represented as mean and standard deviation (SD).

To evaluate the diagnostic ability of the two miRNAs, a receiver operating characteristic (ROC) analysis was performed using SPSS 26 software; briefly, we ran a binary logistic regression to obtain the probability and then ran a ROC curve using the probability as a test variable. The Area under the curve (AUC) quantified the probability that the prediction will be correct after the test variable is observed. For all analyses, significance was set at *p* < 0.05.

### Electronic supplementary material

Below is the link to the electronic supplementary material.

Supplementary Material 1

Supplementary Material 2

Supplementary Material 3

Supplementary Material 4

Supplementary Material 5

Supplementary Material 6

## Data Availability

The RNA-seq raw data discussed in this article have been submitted to Gene Expression Omnibus (GEO; https://www.ncbi.nlm.nih.gov/geo/) with the record GSE243874.
