# Supplementary material for: An RNA-seq study in Friedreich ataxia patients identified hsa-miR-148a-3p as a putative prognostic biomarker of the disease
Source: Hum Genomics. 2024 May 22;18:50. doi: 10.1186/s40246-024-00602-y (PMC11110315; doi:10.1186/s40246-024-00602-y)
Supplement: Supplementary file 2 — Supplementary Material 2 [file 40246_2024_602_MOESM2_ESM.docx]

**Legends to Supplementary Figures**


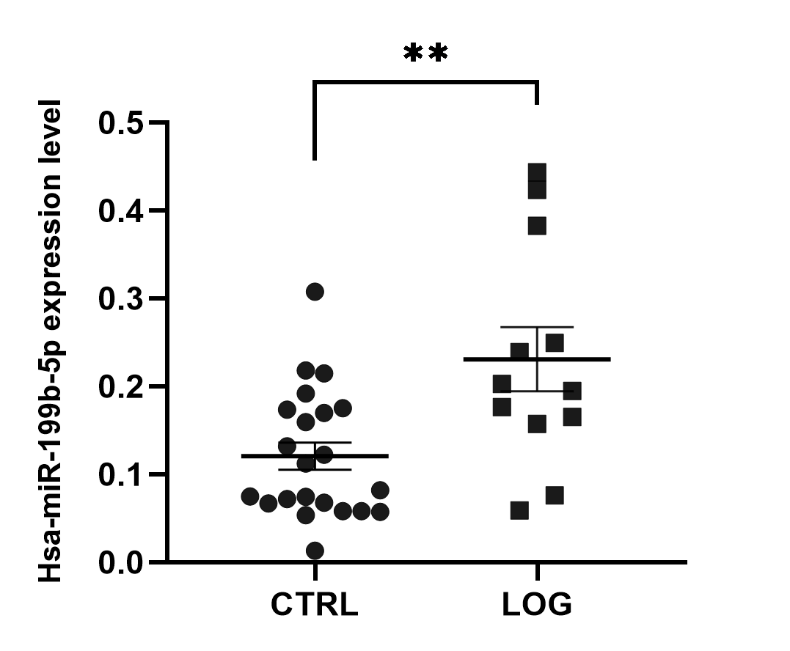


Figure S1. Heatmap showing the expression profiles (scaled TMM) of the differentially expressed sncRNAs in the comparison EOG vs CTRL. The bar on the left visualizes the expression level of the top 5% expressed sncRNAs; in orange-red the upregulated sncRNAs, in blue the downregulated ones.


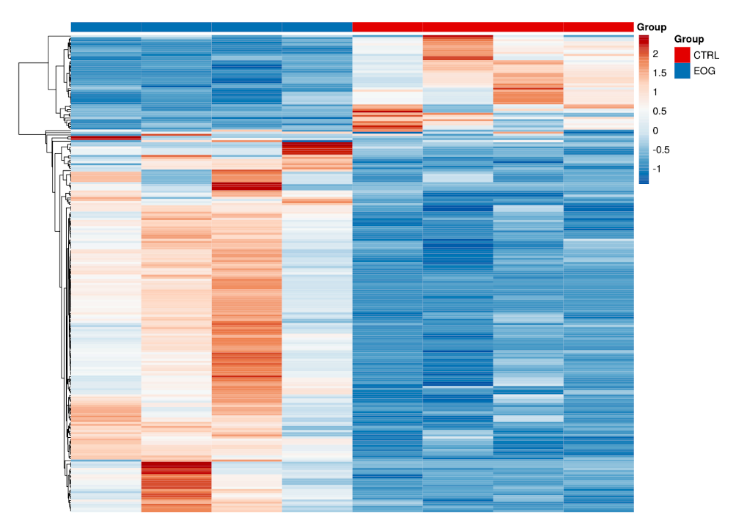


Figure S2. Heatmap showing the expression profiles (scaled TMM) of the differentially expressed sncRNAs in the comparison IOG vs CTRL. The bar on the left visualizes the expression level of the top 5% expressed sncRNAs; in orange-red the upregulated sncRNAs, in blue the downregulated ones.


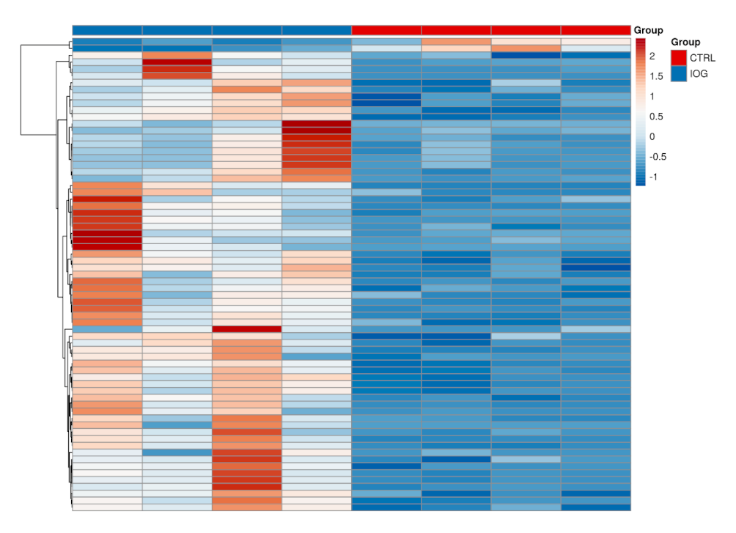

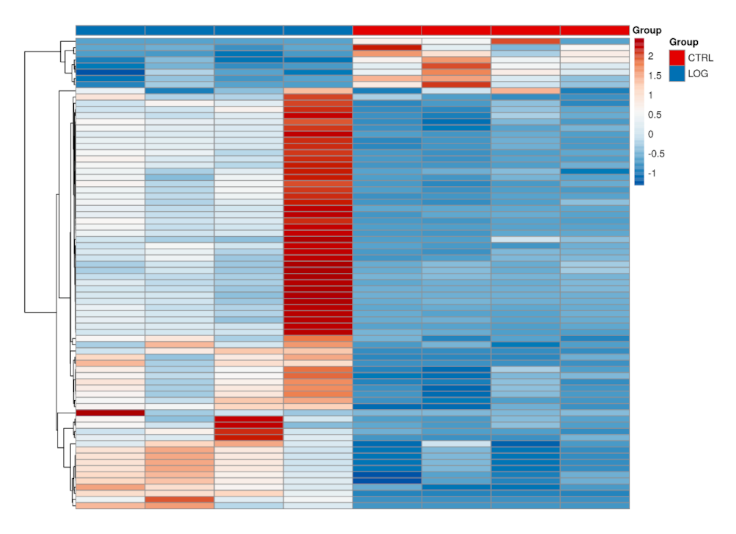


Figure S3. Heatmap showing the expression profiles (scaled TMM) of the differentially expressed sncRNAs in the comparison LOG vs CTRL. The bar on the left visualizes the expression level of the top 5% expressed sncRNAs; in orange-red the upregulated sncRNAs, in blue the downregulated ones.


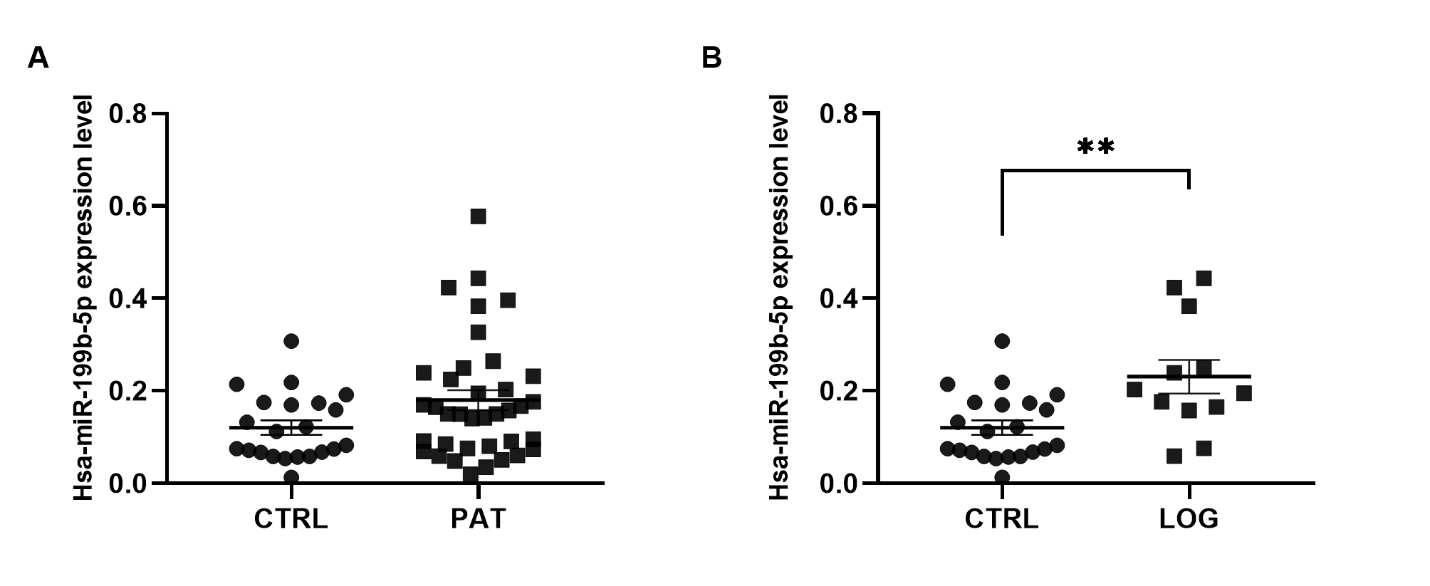


Figure S4. Hsa-miR-199b-5p expression level in plasma of FRDA patients (n = 38). A) Comparison between control subjects (CTRL) and FRDA patients (PAT). Mann-Whitney test. B) Hsa-miR-199b-5p upregulation in Late-Onset FRDA patients Group (LOG) compared to control subject (p < 0.01). Mann-Whitney test.
